# Genetics of Type 2 Diabetes and Clinical Utility

**DOI:** 10.3390/genes6020372

**Published:** 2015-06-23

**Authors:** Rajkumar Dorajoo, Jianjun Liu, Bernhard O. Boehm

**Affiliations:** 1 Genome Institute of Singapore, Agency for Science, Technology and Research, 138672, Singapore; E-Mails: dorajoor@gis.a-star.edu.sg (R.D.); liuj3@gis.a-star.edu.sg (J.L.); 2 Saw Swee Hock School of Public Health, National University of Singapore and National University Health System, Singapore 117549, Singapore; 3 Lee Kong Chian School of Medicine, Nanyang Technological University, Singapore 308232, Singapore; 4 Imperial College London, London, SW7 2AZ, UK; 5 Department of Endocrinology, Tan Tock Seng Hospital, Singapore 308433, Singapore

**Keywords:** type 2 diabetes, genetics, clinical utility

## Abstract

A large proportion of heritability of type 2 diabetes (T2D) has been attributed to inherent genetics. Recent genetic studies, especially genome-wide association studies (GWAS), have identified a multitude of variants associated with T2D. It is thus reasonable to question if these findings may be utilized in a clinical setting. Here we briefly review the identification of risk loci for T2D and discuss recent efforts and propose future work to utilize these loci in clinical setting—for the identification of individuals who are at particularly high risks of developing T2D and for the stratification of specific health-care approaches for those who would benefit most from such interventions.

## 1. Introduction

Diabetes mellitus is recognized as a public health problem of pandemic proportions [1]. Data compiled by the World Health Organization (WHO) in 2014 indicated that the global prevalence of diabetes was close to 10% among adults aged 18 years and above [2]. If present trends persist, projection estimates ominously predict that over half a billion people will develop diabetes within the next two decades, with Asian countries contributing more than 60% of the world’s diabetic population [3]. Hyperglycemia has a pathogenic role in micro-vascular diseases (nephropathy, retinopathy and neuropathy) and is accelerating macro-vascular complications (cardiovascular disease (CVD) such as stroke and coronary heart disease) associated with diabetes. In fact, CVD is the leading cause of premature death among individuals with diabetes. The diabetes pandemic is threatening to severely impact upon and overwhelm healthcare systems in both developed and developing nations and there is an urgent need to curb escalating levels.

Type 2 diabetes (T2D) accounts for approximately 90% of all diabetes patients. Several risk factors for T2D have been identified such as age, sex, central obesity, low physical activity and an unhealthy diet consisting of high saturated fatty acids and/or trans fatty acids and low dietary fiber [4,5]. Prevalence and severity of T2D levels also correlates with ethnicity and certain ethnic groups tend to be more predisposed than others living in similar “obesogenic” environments [6,7]. The “thrifty gene hypothesis” [8] was proposed to explain these observations and suggested that genetic variants that promote metabolic thriftiness may have influenced adaptation of man during an evolution through periods of feast and famine.

Twin and familial studies indicate a substantial heritable component to T2D, estimated to be between 40% and 80% [9,10,11,12]. The initial evidence that T2D in humans may be genetically driven arose through familial linkage studies with the identification of deleterious mutations in genes that caused an early-onset, non-obese, and non-auto-immune form of diabetes, known as maturity-onset diabetes of the young (MODY) [13]. To date, at least 28 distinct genes have been identified to cause monogenic forms of diabetes but being rare events, even in aggregate, monogenic variants affect less than 5% of all diabetes patients [14].

The common form of T2D however, is an archetype multi-factorial disease that arises due to a multitude of genetic variants that may interact with lifestyle and environmental factors (Gene × Environment) [15,16]. Knowledge on the types of inherent gene defects carried by an individual could serve to stratify for those who are at particularly high-risk of developing T2D and stratify specific health-care approaches for individuals who would benefit most from such interventions. A precision medicine approach to tackle diabetes will be of particular interest because the increase in diabetes prevalence is mostly driven by an increasing proportion of young people with the disease [17].

In this review we summarize the recent identification of genetic variants involved in T2D and comment on the use of these in risk prediction and clinical management of patients.

## 2. Genetics of Type 2 Diabetes

With a hypothesis that complex diseases such as T2D may harbor major genetic mutations that are severe enough to cause disease under specific exposures, initial studies in the late 1990s and early 2000s had utilized family linkage studies to evaluate for co-segregation of genetic markers in multiplex families with T2D. Although much effort was invested in these studies the only major success was the identification of the transcription factor 7-like 2 gene (*TCF7L2*) and most of the other putative candidates remain to be validated [18].

In more recent times, advances in technology for SNP genotyping, exploitations of recent genetic knowledge gained from the Human Genome Project and development of robust statistical methods have allowed genome-wide association studies (GWAS) to emerge as the method of choice for detecting common genetic variants associated with complex diseases such as T2D. The first GWAS for T2D, conducted in 2007, with a discovery of about 600 case and control subjects of European ancestry validated the association of *TCF7L2* variants to T2D predisposition and identified novel associations with variants at solute carrier family 30 member 8 (*SLC30A8*) and hematopoietically expressed homeobox (*HHEX*) [19]. One important finding from the initial GWAS results was that effect sizes for common variants involved in T2D were likely to be modest. As such, discovery of additional common variants of similar or even smaller effects were likely to be dependent on larger sample sets that would enable increased power for detecting associations. This led to an innovative data merging strategy now known as GWAS meta-analysis and resulted in multiple waves of GWAS studies for T2D. At least 15 high-profile studies on T2D GWAS study results have been published [19,20,21,22,23,24,25,26,27,28,29,30,31,32,33]. Each newer study with larger sample sets (or those using non-European data or more recent trans-ethnic meta-analysis data) generally corroborated results of preceding studies and at the same time reported additional novel loci associated with the disease. To date, about 80 distinct genetic loci have been identified as predisposing to risks of T2D in European populations through a case-control design and these are summarized in Table 1. Multiple other GWAS efforts have also been invested in evaluating for related quantitative traits, such as blood glucose and insulin levels as well as T2D in non-European populations and these have been summarized and reviewed extensively by others [34,35,36].

**Table 1:** List of identified common variants associated with Type 2 diabetes in populations of European ancestry utilizing a case-control design. Effect estimates drawn from European data from Mahajan *et al.*, 2014 [33] or largest GWAS study.

Location	Reported Gene(s)	SNP	Risk Allele	Other Allele	Risk Allele Frequency	OR (95% CI)
10q25.2	*TCF7L2*	rs7903146	T	C	0.30	1.40 (1.35–1.46)
9q21.31	*TLE4*	rs17791513	A	G	0.93	1.21 (1.13–1.31)
6p22.3	*CDKAL1*	rs7756992	G	A	0.26	1.20 (1.16–1.25)
9p21.3	*CDKN2A, CDKN2B*	rs10811661	T	C	0.82	1.18 (1.13–1.24)
8q24.11	*SLC30A8*	rs3802177	G	A	0.70	1.16 (1.11–1.22)
12q14.3	*HMGA2*	rs2261181	T	C	0.09	1.16 (1.10–1.23)
3p25.2	*PPARG*	rs1801282	C	G	0.88	1.16 (1.10–1.23)
10q23.33	*HHEX, IDE*	rs1111875	C	T	0.58	1.15 (1.11–1.19)
2p21	*THADA*	rs10203174	C	T	0.90	1.15 (1.08–1.21)
16q12.2	*FTO*	rs9936385	C	T	0.39	1.13 (1.09–1.18)
3q27.2	*IGF2BP2*	rs4402960	T	G	0.31	1.13 (1.09–1.17)
11q13.4	*ARAP1, CENTD2*	rs1552224	A	C	0.83	1.13 (1.08–1.19)
6p21.2	*KCNK16*	rs1535500	T	G	0.59	1.13 (1.08–1.19)
7p21.2	*DGKB*	rs17168486	T	C	0.19	1.13 (1.07–1.19)
5q13.3	*ZBED3*	rs6878122	G	A	0.25	1.13 (1.07–1.18)
17q12	*HNF1B*	rs4430796	G	A	0.53	1.13 (1.07–1.09)
7p15.1	*JAZF1*	rs849135	G	A	0.52	1.12 (1.08–1.17)
12q24.31	*HNF1A*	rs12427353	G	T	0.77	1.12 (1.07–1.18)
11q14.3	*MTNR1B*	rs10830963	G	C	0.27	1.11 (1.06–1.16)
7q32.3	*KLF14*	rs13233731	G	A	0.49	1.10 (1.06–1.13)
1p12	*NOTCH2*	rs10923931	T	G	0.11	1.10 (1.04–1.17)
1p32.3	*FAF1*	rs17106184	G	A	0.92	1.10 (1.07–1.14)
8p11.21	*ANK1*	rs516946	C	T	0.77	1.10 (1.06–1.15)
13q31.1	*SPRY2*	rs1359790	G	A	0.73	1.10 (1.05–1.14)
3p24.3	*UBE2E2*	rs7612463	C	A	0.87	1.10 (1.04–1.16)
10q22.3	*ZMIZ1*	rs12571751	A	G	0.51	1.09 (1.06–1.13)
4p16.1	*WFS1*	rs4458523	G	T	0.59	1.09 (1.06–1.13)
3q21.1	*ADCY5*	rs11717195	T	C	0.78	1.09 (1.05–1.14)
12q21.1	*TSPAN8*	rs7955901	C	T	0.47	1.09 (1.05–1.13)
15q25.1	*ZFAND6*	rs11634397	G	A	0.64	1.09 (1.05–1.13)
2q36.3	*IRS1*	rs2943640	C	A	0.63	1.09 (1.05–1.13)
11p15.4	*KCNQ1*	rs163184	G	T	0.50	1.09 (1.04–1.13)
12p11.22	*KLHDC5*	rs10842994	C	T	0.80	1.09 (1.04–1.13)
15q26.1	*PRC1*	rs12899811	G	A	0.30	1.09 (1.04–1.13)
2p16.1	*BCL11A*	rs243088	T	A	0.46	1.09 (1.04–1.13)
4q31.3	*TMEM154*	rs6813195	C	T	0.72	1.08 (1.06–1.10)
15q24.3	*HMG20A*	rs7178572	G	A	0.70	1.08 (1.04–1.13)
11p15.1	*KCNJ11*	rs5215	C	T	0.38	1.08 (1.04–1.12)
1q32.3	*PROX1*	rs2075423	G	T	0.66	1.08 (1.04–1.12)
8q22.1	*TP53INP1*	rs7845219	T	C	0.53	1.08 (1.04–1.12)
18q21.32	*MC4R*	rs12970134	A	G	0.27	1.08 (1.03–1.12)
2p25.3	*TMEM18*	rs10190052	C	T	0.88	1.07 (1.04–1.10)
10q22.1	*C10orf35*	rs2812533	C	T	0.83	1.07 (1.04–1.09)
3q27.3	*LPP*	rs6808574	C	T	0.60	1.07 (1.04–1.09)
6p21.33	*POU5F1, TCF19*	rs3132524	G	A	0.74	1.07 (1.04–1.09)
9q21.32	*TLE1*	rs2796441	G	A	0.63	1.07 (1.03–1.12)
20q13.12	*HNF4A*	rs4812829	A	G	0.16	1.07 (1.01–1.12)
5q11.2	*ARL15*	rs702634	A	G	0.71	1.06 (1.04–1.09)
8q24.21	*TMEM75*	rs1561927	C	T	0.23	1.06 (1.04–1.09)
12q24.31	*MPHOSPH9*	rs1727313	C	T	0.24	1.06 (1.04–1.08)
13q12.13	*RNF6*	rs10507349	G	A	0.74	1.06 (1.04–1.08)
6p21.1	*VEGFA*	rs9472138	T	C	0.24	1.06 (1.04–1.08)
6p24.3	*SSR1, RREB1*	rs9502570	A	G	0.30	1.06 (1.04–1.08)
10q23.31	*PTEN*	rs10788575	A	G	0.16	1.06 (1.03–1.08)
15q22.2	*C2CD4A*	rs7163757	C	T	0.56	1.06 (1.02–1.11)
19q13.32	*GIPR*	rs8108269	G	T	0.30	1.06 (1.02–1.11)
10p13	*CDC123*	rs11257655	T	C	0.23	1.06 (1.01–1.11)
7p14.3	*CRHR2*	rs2284219	T	C	0.32	1.05 (1.03–1.08)
10q26.13	*PLEKHA1*	rs10510110	C	T	0.41	1.05 (1.03–1.07)
1q41	*LYPLAL1*	rs2820446	C	G	0.71	1.05 (1.03–1.07)
5q31.1	*PCBD2*	rs319598	C	T	0.53	1.05 (1.03–1.07)
6q22.32	*C6orf173*	rs4273712	G	A	0.25	1.05 (1.03–1.07)
7p21.2	*ETV1*	rs7795991	G	A	0.54	1.05 (1.03–1.07)
9p24.2	*GLIS3*	rs7041847	A	G	0.50	1.05 (1.01–1.09)
6q23.3	*IL20RA*	rs6937795	A	C	0.42	1.04 (1.02–1.06)
15q26.1	*AP3S2*	rs2028299	C	A	0.29	1.04 (1.00–1.09)
2q24.3	*GRB14*	rs3923113	A	C	0.61	1.04 (1.00–1.09)
3p14.1	*PSMD6*	rs831571	C	T	0.81	1.03 (0.99–1.08)
3q27.3	*ST64GAL1*	rs16861329	C	T	0.85	1.03 (0.96–1.10)
10q22.1	*VPS26A*	rs1802295	T	C	0.33	1.02 (0.98–1.06)
15q14	*RASGRP1*	rs7403531	T	C	0.22	1.02 (0.98–1.06)
19q13.11	*PEPD*	rs3786897	A	G	0.57	1.02 (0.98–1.06)

## 3. Clinical Utility of Identified Genetic Variants

The advent of GWAS era and the identification of multiple risk loci have no doubt illuminated the pathophysiology of T2D. These studies have confirmed the polygenic nature of the disease and interestingly implicate a larger number of hits to beta-cell function (insulin secretion) as opposed to those involved in insulin resistance [34,35,36]. The identification of these loci has also very recently provided an opportunity to translate genetic information to clinical practice. These may have potential roles in disease risk prediction—to identify subjects at risk of developing disease at an early-stage, and in clinical management of individuals—to tailor treatment regimes so that affected individuals would benefit most by leveraging on the so-called legacy effect, *i.e.*, early tight diabetes control resulting in a substantial micro- and cardiovascular benefit [37,38].

## 4. Utility of Genetic Variants in T2D Risk Prediction

Using identified genetic variants, studies have attempted to predict undiagnosed individuals with T2D using cross-sectional studies and incident T2D using longitudinal studies. Early studies such as those done by the Diabetes Prevention Program (DPP) provided much optimism and showed that common variants at the *TCF7L2* locus predict the progression to diabetes in subjects with impaired glucose tolerance [39]. However, available data to date, in aggregate, do not provide robust evidence to support the utility of genetic screens (composed of recently identified genetic variants) for T2D predictions. The discriminatory ability of identified genetic variants in cross-sectional studies indicate a modest area under the receiver operating characteristic curve (AUC), of approximately 0.60, with mixed significance levels when combined with traditional clinical models (e.g., age, sex, obesity, family history and fasting blood glucose levels) [40,41,42,43]. Longitudinal prospective studies also report similarly unconvincing and mixed results of T2D predictions [4,44,45].

Before concluding that genetic screening for the risk of T2D is completely futile, it would be important to address the limitations of these prediction studies. One primary limitation would be that of modest effect sizes of common variants, especially those identified through recent GWAS for T2D. The initial *TCF7L2* variant still remains the strongest common variant identified to date (each copy of the T allele of rs7903416 carries 1.4 increased odds of T2D (Table 1)) and even in combination all identified variants only explain less than 15% of the heritability of T2D [32]. It is also crucial to place current prediction data in the context of the heterogenous nature of the disease. For example, T2D risk prediction has been reported to be improved among younger subjects as compared to older subjects (below 50 years old *vs.* above 50 years old) [45]. Most prediction models of prospective studies have been performed utilizing subjects of a limited age range (primarily subjects > 30 years old) and follow-up years (approximately 10 years or less). A longer time horizon is likely to improve the predictive value of genetic variants relative to other T2D risk predictors (such as obesity and blood glucose) that can vary with time. Furthermore, it is difficult to precisely define T2D, other than chronic hyperglycemia that is not explained by Type 1 diabetes, monogenic or syndromic forms of the disease, gestational diabetes or drug and chemical induced diabetes [46]. Thus, T2D is likely to encompass a cluster of several disease subtypes resulting from defects in varied pathways. The identification of latent autoimmune diabetes in adults (LADA) and increasing number of monogenic forms of diabetes may also imply a level of misclassification that is likely to affect accuracy of prediction models [47,48]. It is also noteworthy that there is a research gap on incorporation of Gene × Gene and Gene × Environmental interactions in prediction models. It can, thus, be anticipated that enhanced precision in T2D diagnosis and improved risk prediction models will more accurately reveal the true potential of genetic screens in disease prediction. With continued efforts to further characterize identified variants (e.g., fine-mapping studies) and unearth additional genetic variants missed by GWAS (e.g., rare variants, coding variants and structural variants) it is likely that a full complement of genetic variants could be evaluated and utilized for disease predictions in the near future.

## 5. Utility of Genetic Variants in Clinical Management

Tight glycemic control is fundamental in the clinical management of diabetes. Complications of diabetes (both micro-vascular and macro-vascular) primarily arise due to chronic, uncontrolled hyperglycemia. Study results of three seminal trials conducted in the 1990s—Diabetes Control and Complication Trial (DCCT), UK Prospective Diabetes Study (UKPDS) and the Kumamoto study [49,50,51,52] showed that early and aggressive glycemic control significantly reduced rates of micro-vascular complications. Results from longer follow-ups also demonstrated improvements in macro-vascular CVD events and in overall mortality levels [53,54]. There is substantial evidence of a “legacy effect”, arising from aggressive treatment regimes to tightly control blood glucose early in the natural history of the disease, which subsequently reduces the risks of diabetes complications and improves patient outcome.

More recent intervention studies such as the Action to Control Cardiovascular Risk in Diabetes (ACCORD), Action in Diabetes and Vascular Disease: Preterax and Diamicron Magnetic Resonance Controlled Evaluation (ADVANCE) and the Veterans’ Administration Diabetes Trial (VADT) have further corroborated the importance of early, aggressive treatment regimes for diabetes [55,56,57,58]. However, these studies also demonstrate additional factors that may influence outcomes such as age, obesity status, diabetes duration and number of existing co-morbidities. This has called for treatment such as choice of therapeutics and levels of blood glucose reductions to be individualized so that optimal patient outcomes can be met [59,60,61].

It is reasonable to question if recent genetic findings can help to stratify patients so that clinical management can be individualized. Early studies have shown that *TC7L2* risk allele carriers were less likely to respond to sulfonylureas but not to metformin [62]. Studies have also highlighted potential roles of variants in multidrug and toxin extrusion 1 (*MATE1*) and ataxia telangiectasia mutated (*ATM*) genes that may affect the effectiveness of metformin treatments [63,64]. A recent clinical trial demonstrated the potential application of utilizing a genetic risk variant (rs553668 at adrenoreceptor alpha 2A (ADRA2A)) to guide therapeutic interventions using the α-2A adrenergic receptor antagonist yohimbine [65]. Another very recent pharmacogenomic study also showed that efficacy of newer generation therapeutics, such as linagliptin that enhances glucose homeostasis in diabetics by improving incretin response, was also influenced by the number of *TCF7L2* risk variants [66]. The same study also reported a lack of a more pronounced effect among homozygous risk allele carriers (TT genotype compared to CC genotype of rs7903146) and it is suggested that instead of utilizing single variants, it may be possible that the full complement of known risk variants might be evaluated together with additional novel variants from pharmacogenetic studies to understand the combined effect inherent genetics plays in influencing therapeutic response. In this respect, there is a wide research gap in the field and continued efforts may uncover the potential of individualizing diabetes therapeutic regimes based on inherent genetics.

## 6. Future Directions and Conclusions

Paradigm shifts in the future of diabetes medicine are required if current escalating trends are to be curbed. Diabetes management and/or prevention should aim to incorporate genetic and molecular screens to tailor specific treatments (therapeutics as well as lifestyle changes) and optimize benefits for patients. The translation of this vision to clinical practice is likely to depend on a more thorough phenotyping combined with genotyping of individuals at an early-stage (pre-diabetes) so that disease subtypes can be identified, appropriate treatment regimes can be specifically selected subtypes and the “legacy effect” of initial treatment can be fully exploited.

Now that genetic testing for MODY has been made routine and can affect diagnosis and treatment of affected individuals [67], some progress has already been observed with more extensive characterization of disease states. MODY subjects with deleterious hepatocyte nuclear factor 1-alpha (*HNF1A*) gene mutations are often misdiagnosed as having T2D and importantly, are known to respond more effectively to sulphonylureas than metformin [68,69,70,71,72]. Thus, knowledge on inherent genetics has helped to efficiently manage, on a long-term basis, the hyperglycemia in individuals with this class of the disease. Further large-scale studies on the influence genetic variants exert on various T2D drug classes and additional in-depth characterization of these findings may allow for novel clinical utilization of pharmacogenetics.

Although much progress has been made with recent genetic discoveries for T2D risks, their role in genetic prediction is less clear. As discussed, this is partly due to the relatively small proportion of heritability explained by these variants identified from GWAS [32,33]. The community can brace itself for future studies that will likely lessen this “missing heritability” deficit and are expected to highlight the role of variants missed by GWAS (e.g., rare, coding and structural variants) in T2D predisposition. As large-scale GWAS meta-analyses are by design expected to enrich for pure genetic effects, it is also likely that variants modified by important environmental and life-style factors may have remained unidentified. Future efforts to characterize the role of Gene × Environment interactions as well as other epigenetic modifications can be expected to fill this fissure in diabetes research [16,70]. Lastly, with continued technological advancements and reduced costs, it has presently become possible to carry out integrated assessments of various omics (e.g., genomics, transcriptomics, proteomics, metabolomics, microbiomics) in large cohorts to get a holistic view of disease states [71,72,73,74,75]. These future efforts can be expected to refine the molecular characterization of T2D that may be subsequently evaluated for clinical translation.
